# FOXA1 Leads to Aberrant Expression of SIX4 Affecting Cervical Cancer Cell Growth and Chemoresistance

**DOI:** 10.1155/2022/9675466

**Published:** 2022-04-20

**Authors:** Zhuo Wang, Bao-Sheng Sun, Zhi-Shen Chen, Kang-Kang Zhao, Yun-Long Wang, Fan-Xu Meng, Yang Zhang

**Affiliations:** ^1^Department of Radiotherapy, Jilin Cancer Hospital, Changchun, 130000 Jilin, China; ^2^Department of Oncology, Jilin Cancer Hospital, Changchun, 130000 Jilin, China

## Abstract

Cervical cancer (CC) is among the most prevalent cancers among female populations with high recurrence rates all over the world. Cisplatin (DDP) is the first-line treatment for multiple cancers, including CC. The main problem associated with its clinical application is drug resistance. This study is aimed at investigating the function and downstream regulation mechanism of forkhead-box A1 (FOXA1) in CC, which was verified as an oncogene in several cancers. Using GEO database and bioinformatics analysis, we identified FOXA1 as a possible oncogene in CC. Silencing of FOXA1 inhibited CC cell growth, invasion, and chemoresistance. Afterwards, the downstream gene of FOXA1 was predicted using a bioinformatics website and validated using ChIP and dual-luciferase assays. SIX4, a possible target of FOXA1, promoted CC cell malignant aggressiveness and chemoresistance. In addition, overexpression of SIX4 promoted phosphorylation of PI3K and AKT proteins and activated the PI3K/AKT signaling pathway. Further overexpression of SIX4 reversed the repressive effects of FOXA1 knockdown on CC cell growth, invasion, and chemoresistance in DDP-resistant cells. FOXA1-induced SIX4 facilitates CC progression and chemoresistance, highlighting a strong potential for FOXA1 to serve as a promising therapeutic target in CC.

## 1. Introduction

Despite as one of the most preventable malignancies through screening, cervical cancer (CC) claimed the lives of 4138 women in 2018 in the United States, and one-half of whom were aged ≤58 years at death [1]. Treatment for CC patients depends on the disease severity at diagnosis and locally available resources and may include radical hysterectomy or chemoradiation or a combination of both [2]. The chemotherapeutic agent cisplatin (DDP), a small-molecule platinum compound that was firstly identified to inhibit bacterial growth and later identified as an anticancer drug, has been applied to effectively treat advanced/recurrent CC [3]. The acquirement of DDP resistance in human cancer cells *in vivo* and *in vitro* stems from complex genetic and epigenetic alterations in gene expression [4].

Forkhead-box A1 (FOXA1) is expressed by preinvasion lesions of the uterine cervix and almost half invasive CC, supporting its implication in human papilloma virus pathogenesis [5]. Moreover, FOXA1 knockdown in DDP-resistant nasopharyngeal carcinoma cells increased the chemosensitivity to DDP [6]. Therefore, we wondered there is also an association between FOXA1 overexpression and DDP resistance in CC cells. Interestingly, FOXA1 was confirmed to induce the expression of circRNA derived from oxysterol binding protein-like 10 in CC cells, thereby regulating CC cell proliferation and migration [7]. As a consequence, we believed that the possible effects of FOXA1 on DDP resistance in CC are elicited through its transcription factor role. The sine oculis homeobox (SIX) family homeobox genes have been displayed to participate in the tumor initiation and progression [8]. The functions of SIX genes are predominantly based on their structure and their regulatory roles in response to external or internal stimuli, which suggests that members of the SIX superfamily can be considered as new target molecules to inhibit tumor growth and progression [9]. For one of them, SIX1 was induced by the E7 oncoprotein of human papillomaviruses in CC, and increased SIX1 expression contributed to the upregulation of genes related to the initiation of DNA replication [10]. In the present study, SIX4 was identified as a downstream target of FOXA1 using a bioinformatics tool hTFtarget (http://bioinfo.life.hust.edu.cn/hTFtarget#!/). SIX4 has been lately revealed to indicate dismal clinical outcome of patients with esophageal squamous cell carcinoma and to potentiate tumor growth and cell metastasis [11]. However, its role in CC, especially in regulating chemoresistance, remains largely unclear. Hence, our study investigated the potential roles of FOXA1 in development of CC and its functions in connection with SIX4.

## 2. Materials and Methods

### 2.1. Cell Culture

Normal cervical epithelial cells H8 were from Tongpai (Shanghai, China). Human CC cells HT-3 (HTB-32), CaSki (CRL-1550), HeLa (CCL-2), and SiHa (HTB-35) were from ATCC (Manassas, VA, USA). The cells were grown in Roswell Park Memorial Institute- (RPMI-) 1640 medium (A1049101, Thermo Fisher Scientific, Waltham, MA, USA) plus 10% FBS, 100 U/mL penicillin, and 100 *μ*g/mL streptomycin sulfate and placed in an incubator at 37°C and 5% CO_2_ until cells were stable.

HT-3 and HeLa cells were incubated with DDP (P4394, Sigma-Aldrich Chemical Company, St Louis, MO, USA) at different concentrations. DDP-resistant HT-3 cells (HT-3/DDP) and HeLa-resistant cells (HeLa/DDP) were initially established in our laboratory from parental HT-3 and HeLa cells by exposure to progressively increasing concentrations of DDP over a 6-month period. Subsequently, 3-(4,5-dimethylthiazol-2-Yl)-2,5-diphenyltetrazolium bromide (MTT) assays were conducted to determine the successful development of drug-resistant cell lines.

### 2.2. Cell Transfection

The short hairpin RNA targeting FOXA1 (sh-FOXA1), SIX4 overexpression vector (oe-SIX4), and their respective controls used for HT-3/DDP and HeLa/DDP cell transfection were purchased from Shanghai GenePharma Co., Ltd. (Shanghai, China). When cell confluence reached 90%, transfection was then performed using Lipofectamine 3000 (L3000015, Invitrogen, Carlsbad, CA, USA) as per the manufacturer's instructions.

### 2.3. Complementary DNA (cDNA) Synthesis and RT-qPCR

Total RNA was isolated from cells using TRIzol (15596018, Thermo Fisher), and the concentration and quality of total RNA were measured using a Nanodrop spectrophotometer (ND-LITE-PR, Thermo Fisher). For mRNA expression analysis, cDNA was synthesized using PrimeScript RT master mix (RR036B, Takara Holdings Inc., Kyoto, Japan). Real-time PCR was performed using SYBR Select Master Mix (4472919, Thermo Fisher Scientific) on a CFX96 real-time PCR detection system (Bio-Rad, Inc., Hercules, CA, USA). Relative expression was calculated utilizing 2^−*ΔΔ*Ct^ method with normalization to glyceraldehyde-3-phosphate dehydrogenase (GAPDH). The sequences of primers are presented in Table 1.

### 2.4. Western Blot

Total protein in the cells was extracted using radioimmunoprecipitation assay buffer (R0010, Solarbio, Beijing, China) and quantified using a protein bicinchoninic acid analysis kit (71285-3, Sigma). Protein lysates were separated by sodium dodecyl sulfate-polyacrylamide gel electrophoresis and transferred to PVDF membranes (3010040001, Millipore, Billerica, MA, USA). After being sealed in 5% skim milk for 120 min at room temperature, the membranes were treated with specific primary antibodies overnight at 4°C and with the secondary antibody for 120 min at room temperature. Protein bands were detected with enhanced chemiluminescence solution (PE0010, Solarbio) and imaged with a GelDoc Go system (Bio-Rad, Hercules, CA, USA). Relative expression of proteins was measured using ImageJ with GAPDH as an internal reference. The antibodies used in the experiments are as follows: primary antibodies were FOXA1 (1 : 1000, ab170933, Abcam, Cambridge, MA, USA), SIX4 (1 : 2000, ab176713, Abcam), GAPDH (1 : 2000, GTX124502, GeneTex, Inc., Alton Pkwy Irvine, CA, USA), p-PI3K (1 : 1000, PA5-118549, Thermo Fisher Scientific), PI3K (1 : 1000, #4257S, Cell Signaling Technologies, Beverly, MA, USA), p-AKT (1 : 1000, ab38449, Abcam), and AKT (1 : 500, ab8805, Abcam); secondary antibody was goat anti-rabbit IgG antibody (ab6721, 1 : 2000, Abcam).

### 2.5. Colony Formation Assay

HT-3/DDP and HeLa/DDP cell suspensions were seeded into 6-well plates at approximately 5 × 10^2^ cells/well. Subsequently, 2 mL RPMI-1640 medium was supplemented to each well, and the solution was changed at an interval of 2 d. After 10 d, the cells were fixed with formaldehyde and treated with crystal violet, and the number of colonies formed in each well of the plate was counted.

### 2.6. 5-Ethynyl-2′-Deoxyuridine (EdU) Assay

The EdU Staining Proliferation kit (ab222421, Abcam) was applied to assess the proliferation of HT-3/DDP and HeLa/DDP cells, and all operations were performed strictly as per the instructions. Stably transfected cells (5 × 10^3^ cells/well) were seeded into 96-well culture dishes and grown for 24 h. Then, 100 *μ*L medium containing 20 *μ*M EdU was supplemented into each well, and the cells were incubated at 37°C for 2 h. The number of EdU-positive cells was observed by fluorescence microscopy (Olympus Optical Co., Ltd., Tokyo, Japan) and counted.

### 2.7. Transwell Assay

A 24-well plate and 8 *μ*m Transwell cell culture chambers (Corning Glass Works, Corning, NY, USA) coated with Matrigel were used for Transwell experiments to determine the invasion of cells. Briefly, 1 × 10^5^ cells in 200 *μ*L serum-free medium were placed in the apical chamber. RPMI-1640 medium (Thermo Fisher) containing 10% FBS was supplemented to the basolateral chamber. After 24 h, a cotton swab was used to remove the noninvaded cells, and the membranes were fixed with 10% neutral formalin buffer (SL1570, Coolaber, Beijing, China) for 30 min and stained with 0.5% crystal violet (C0121, Beyotime Biotechnology, Shanghai, China) for 10 min. The cells were observed under a microscope (Olympus), and five fields were randomly selected to count the number of invaded cells.

### 2.8. MTT Assay

The MTT kit was utilized to detect the viability of the cells. Briefly, HT-3/DDP and HeLa/DDP cells (2 × 10^4^ cells) were plated in 96-well plates. Different concentrations of DDP solution diluted with dimethylsulfoxide (DMSO, D2650, Sigma-Aldrich) were added to each well separately and incubated for 2 d. For cells in each well, 20 *μ*L MTT solution (5 mg/mL, M2003, Sigma-Aldrich) was added, and the cells were incubated for 4 h in an incubator at 37°C with 5% CO_2_. After removal of the medium, DMSO (100 *μ*L/well) was added to dissolve the formed formazan crystals. The optical density (OD) values were evaluated at 570 nm using a Bio-Rad 550 microplate reader (Bio-Rad).

### 2.9. Dual-Luciferase Assay

A potential binding site for the SIX4 promoter with a conserved FOXA1 binding sequence was obtained from Jaspar (http://jaspar.genereg.net/), and the binding region was PCR amplified and cloned into the pGL3 vector (Promega, Madison, WI, USA) to obtain a Promoter luciferase reporter vector. The above reporter vectors were cotransfected with sh-NC or sh-FOXA1 into HT-3/DDP and HeLa/DDP cells, respectively, using Lipofectamine 3000 (Invitrogen). After 48 h of transfection, luciferase activity was tested by a dual-luciferase reporter system (Promega).

### 2.10. Chromatin Immunoprecipitation (ChIP)

ChIP was conducted using the EZ-Magna ChIP kit (17-10461, Sigma-Aldrich). Briefly, HT-3 or HeLa cells were cross-linked by using a 1% formaldehyde solution for 10 min at room temperature and then quenched by glycine. DNA fragments were obtained by ultrasound treatment. The lysate was immunoprecipitated with anti-FOXA1 (1 : 50, ab170933, Abcam) or IgG (1 : 100, ab172730, Abcam)-coupled magnetic beads. Finally, the enrichment ability of the precipitates for the SIX4 promoter was analyzed by PCR.

### 2.11. Xenograft Tumor Model

The whole procedures of experiments were approved by the committee of Jilin Cancer Hospital. Twelve BALB/c nude mice (female, 4-6 weeks, 21.2 ± 2.3 g, Vital River, Beijing, China) were housed and cared according to the institutional guidelines for animal care. The mice were randomly divided into 2 groups (sh-NC and sh-FOXA1). A 12-12 h lights on-off cycle was maintained in a specific-pathogen-free environmentally controlled room (25°C, 45% humidity). The mice were given adequate food and water. HeLa/DDP cells transfected with sh-NC or sh-FOXA1 suspended in serum-free RPMI-1640 medium were injected subcutaneously into nude mice after 1 week of acclimation feeding. After that, the tumor volume of the mice was measured every 7 days. Tumor volume = 0.5 × length × width^2^. After 4 weeks, the mice were euthanized by intraperitoneal injection of 1% pentobarbital sodium, and tumors were collected for weighing.

### 2.12. Immunohistochemistry

The tumor tissues of mice were fixed in formalin for 4 h, routinely embedded in paraffin, and sectioned (5 *μ*m). The dewaxed and hydrated tissues were fixed on slides and incubated for 15 minutes at room temperature with 3% H_2_O_2_, followed by the incubation with normal goat serum (SL038, Solarbio) for 15 min at room temperature. The sections were stained with primary antibody KI67 (1 : 100, 14-5699-82, Thermo Fisher scientific) overnight at 4°C and with secondary antibody (1 : 2000, ab205719, Abcam) for 15 min at 37°C. Afterwards, 40 *μ*L HRP-labeled streptavidin working solution (SE068, Solarbio) was added dropwise and incubated for 15 min. After a 30 s hematoxylin counter-staining, dehydration, and fixation, the number of KI67-positive cells in the sections was counted under a light microscope (Olympus), and five areas were randomly selected for each section separately.

### 2.13. Statistical Analysis

The SPSS 22.0 statistic software (SPSS, Inc, Chicago, IL, USA) was used for statistical analyses. The data were presented as mean ± SD. Unpaired *t*-tests were applied to describe the differences between different groups. Data among multiple groups were compared by one-way or two-way ANOVA followed by a Tukey's post hoc test. We considered *p* < 0.05 as significant in all cases.

## 3. Results

### 3.1. FOXA1 Is Highly Expressed in CC and Is Associated with Drug Resistance

**Figure 1:**
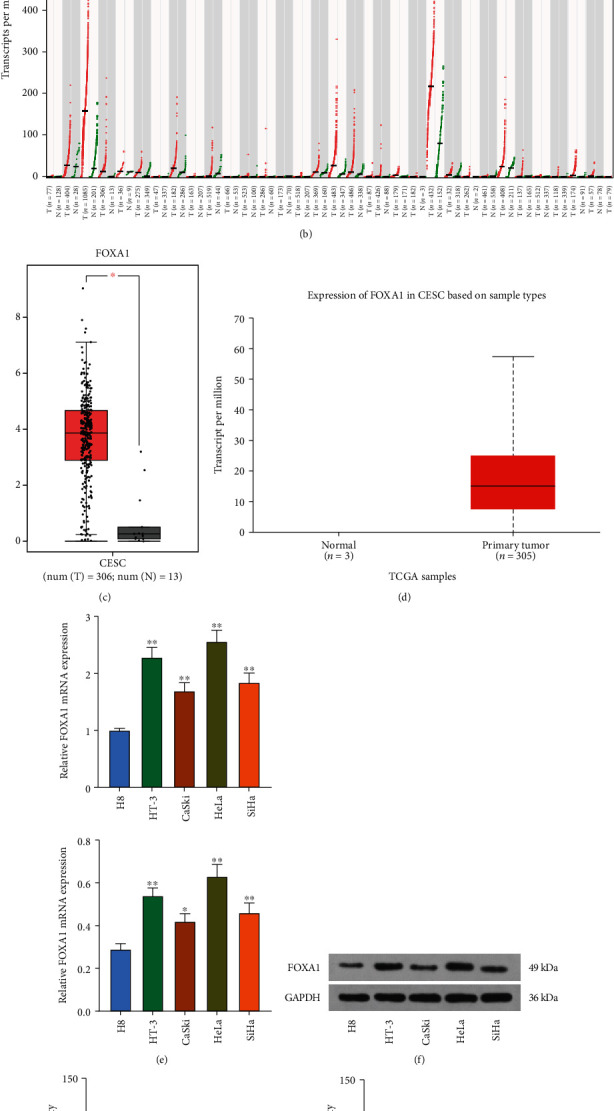
FOXA1 is highly expressed in CC and is associated with drug resistance. (a) GEO database analysis of differentially expressed transcription factors in GSE64217 and GSE63514 datasets and Venn map plotting. (b) Expression of FOXA1 in multiple cancers in the GEPIA database. (c) FOXA1 is highly expressed in TCGA-CESC database. (d) High expression of FOXA1 in CESC in TCGA database. (e, f) FOXA1 expression in normal cervical epithelial cells H8 and CC cell lines (HT-3, CaSki, HeLa, and SiHa) at mRNA and protein levels by (e) RT-qPCR and (f) Western blot. (g) The IC50 value of cells to DDP by MTT assay to confirm the successful establishment of drug-resistant cell lines. (h, i) Detection of FOXA1 expression in parental cells and drug-resistant cells by (h) RT-qPCR and (i) Western blot. Data among multiple groups were analyzed by one-way or two-way ANOVA followed by Tukey's post hoc test (^∗^*p* < 0.05; ^∗∗^*p* < 0.01). The cell experiment was independently repeated three times.

First, we found the gene expression profiles GSE64217 (incisional biopsies from cervical squamous cell carcinoma and normal cervical mucosa) and GSE63514 (24 normal and 28 cancer specimens) regarding CC were downloaded from the GEO database (https://www.ncbi.nlm.nih.gov/geo/). We screened 179 and 192 differentially expressed transcription factors with *p* < 0.05 and log2FC > 1 or log2FC > 2 as screening conditions, respectively. Among the intersecting genes, we screened out that FOXA1 expression was closely associated with CC (Figure 1(a)). By querying the GEPIA database (http://gepia.cancer-pku.cn/), we found that FOXA1 was highly expressed in various cancers (Figure 1(b)) and that FOXA1 was significantly differentially expressed in CC tissues versus normal cervical tissues (Figure 1(c)). Similarly, analysis of TCGA database on FOXA1 expression in CC through the UALCAN website (http://ualcan.path.uab.edu/index.html) revealed that FOXA1 was significantly highly expressed in CC (Figure 1(d)). Consistently, FOXA1 expression was much higher in CC cell lines (HT-3, CaSki, HeLa, and SiHa) than that in normal cervical epithelial cells H8 (Figures 1(e) and 1(f)). Subsequently, we selected HT-3 and HeLa cells with significant differences of FOXA1 expression to induce DDP-resistant HT-3 cell lines (HT-3/DDP) and HeLa cell lines (HeLa/DDP). The successful development of drug-resistant cell lines was validated using the MTT assay (Figure 1(g)). The results of RT-qPCR and Western blot revealed that FOXA1 was upregulated in drug-resistant cells relative to parental cells (Figures 1(h) and 1(i)). The above results suggest that FOXA1 is highly expressed in CC and is associated with drug resistance.

### 3.2. Silencing of FOXA1 Inhibits CC Cell Growth and Chemoresistance

**Figure 2:**
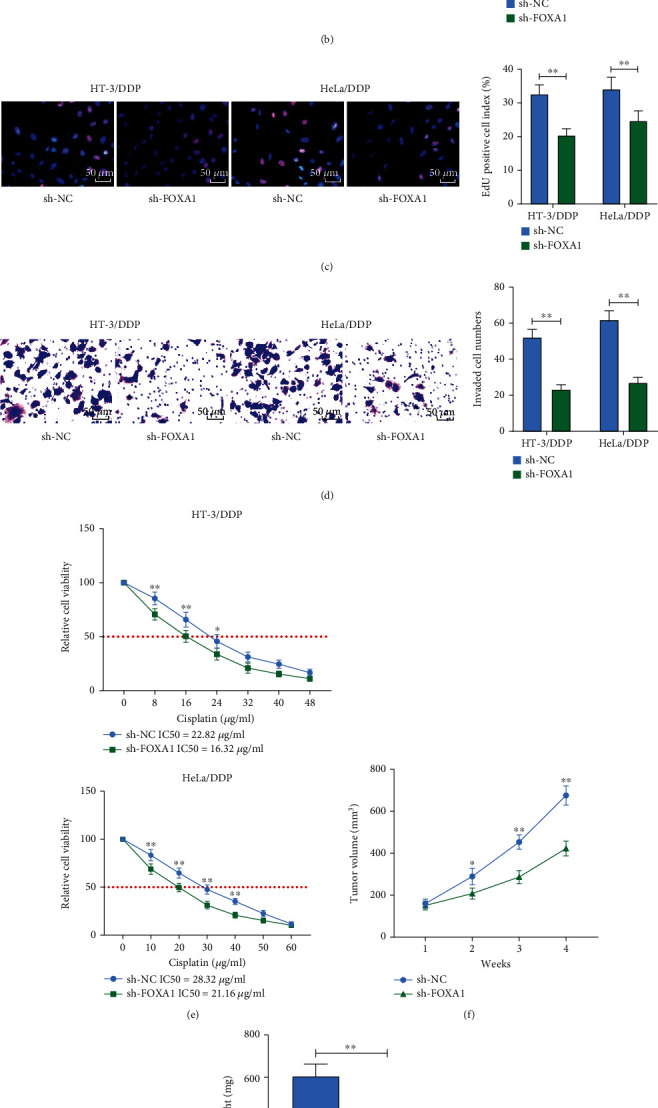
Silencing of FOXA1 inhibits CC cell growth and reduces chemoresistance to DDP. (a) Detection of FOXA1 mRNA expression in HT-3/DDP and HeLa/DDP cells after transfection with sh-NC or sh-FOXA1 by RT-qPCR. (b) The proliferation of cells measured using colony formation assay. (c) The cell viability measured using EdU assay. (d) The invasive ability of cells determined using Transwell assay. (e) The chemoresistance of cells to DDP measured using MTT assay. (f) Effect of knockdown of FOXA1 on tumor volume in nude mice. (g) Effect of knockdown of FOXA1 on tumor weight. (h) Positive rate of KI67 in tumors by immunohistochemistry. Data among multiple groups were analyzed by unpaired *t*-test or two-way ANOVA followed by Tukey's post hoc test (^∗^*p* < 0.05; ^∗∗^*p* < 0.01). The cell experiment was independently repeated three times.

To define the effect of FOXA1 on the growth and drug resistance of HT-3 and HeLa cells, we transfected sh-NC or sh-FOXA1 into HT-3/DDP and HeLa/DDP cells, and the expression of FOXA1 in the cells was detected 48 h posttransfection to confirm the successful transfection (Figure 2(a)). Changes in the activity of drug-resistant cells were detected by colony formation assay and EdU staining, and it was found that the activity of drug-resistant cells was significantly inhibited after knocking down FOXA1 (Figures 2(b) and 2(c)). According to the Transwell assay, we further identified that the invasive ability of drug-resistant cells was significantly reduced after silencing of FOXA1 (Figure 2(d)). MTT assay results showed that chemoresistance to DDP decreased more significantly in the sh-FOXA1 group with increasing concentrations of DDP (Figure 2(e)). Subsequently, in *in vivo* experiments, we selected HeLa/DDP cells with stable low expression of FOXA1 for tumor xenograft experiments. It was demonstrated that loss of FOXA1 significantly hindered the growth of HeLa/DDP cells *in vivo* (Figures 2(f) and 2(g)). Moreover, the positive rate of KI67 in the tumors was significantly reduced after the FOXA1 knockdown (Figure 2(h)).

### 3.3. Knockdown of FOXA1 Downregulates the Expression of SIX4 in CC Cells

**Figure 3:**
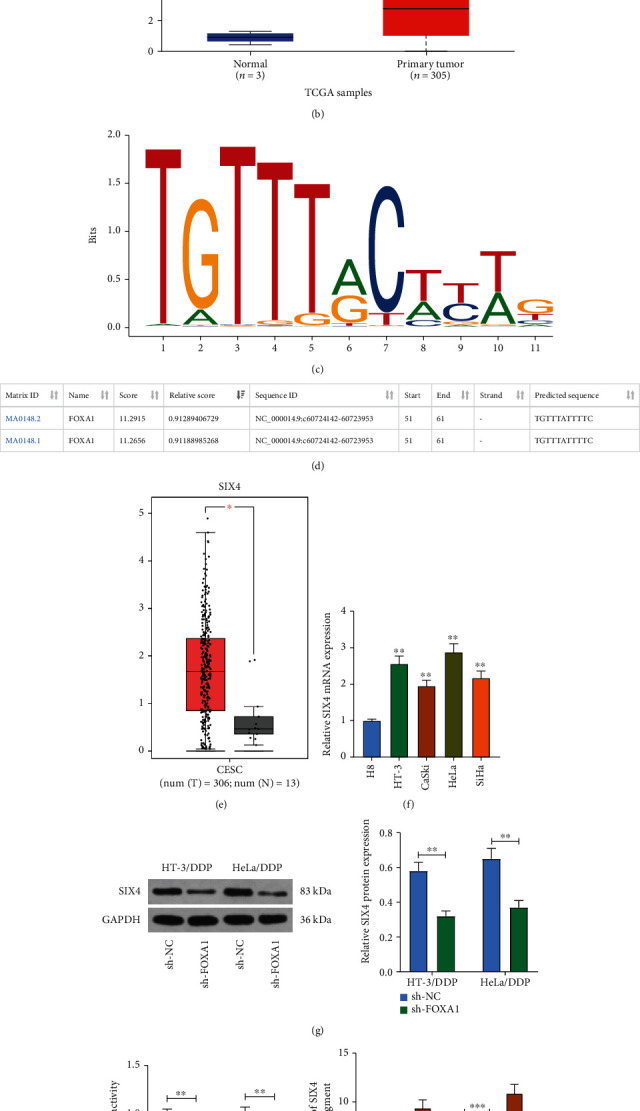
Knockdown of FOXA1 inhibits SIX4 expression in CC cells. (a) SIX4 was predicted to be a downstream gene of FOXA1 using hTFtarget database. (b) High expression of SIX4 in CESC in TCGA database. (c) Conserved binding sites for FOXA1. (d) Potential binding sites of SIX4 promoter to FOXA1. (e) SIX4 expression in TCGA-CESC database. (f) Detection of SIX4 mRNA expression in normal cervical epithelial cells H8 and CC cell lines (HT-3, CaSki, HeLa, and SiHa) by RT-qPCR. (g) Detection of SIX4 protein expression in drug-resistant cells after knockdown of FOXA1 measured using Western blot. (h) The effect of low expression of FOXA1 on the Promoter luciferase activity of SIX4 measured using dual-luciferase assay. (i) The ability of anti-FOXA1 to enrich SIX4 promoter sequence examined using ChIP assay. Data among multiple groups were analyzed by one-way or two-way ANOVA followed by Tukey's post hoc test (^∗^*p* < 0.05, ^∗∗^*p* < 0.01, ^∗∗∗^*p* < 0.001). The cell experiment was independently repeated three times.

To explore the pathways through which FOXA1 acts on CC, the hTFtarget database was queried. Among all the downstream genes of FOXA1, we found that SIX4 had the largest log2FC value (log2FC = 7.11) in the GSE63514 database, so we selected SIX4 as the downstream target of FOXA1 in this study (Figure 3(a)). SIX4 was significantly highly expressed in CC as queried by TCGA database (Figure 3(b)). We then obtained the conserved binding sequence of FOXA1 in Jaspar (Figure 3(c)). The promoter sequence of SIX4 was also predicted to have a potential binding site to FOXA1 (Figure 3(d)). The GEPIA database predicted the overexpression of SIX4 in CC (Figure 3(e)). RT-qPCR result was consistent with the prediction (Figure 3(f)). The SIX4 expression in HT-3/DDP and HeLa/DDP cells with FOXA1 knockdown was detected by Western blot, and its protein expression was found to be significantly constrained (Figure 3(g)). We constructed the Promoter luciferase reporter vector by inserting the SIX4 promoter sequence with the highest binding score of potential to FOXA1 into the pGL3 luciferase vector. The vectors were cotransfected with sh-FOXA1 or its control into HT-3/DDP and HeLa/DDP cells, respectively. The luciferase activity was measured after 48 h. Inhibition of FOXA1 was found to significantly suppress the luciferase activity of Promoter (Figure 3(h)). In ChIP assays, we observed that anti-FOXA1 significantly enriched the promoter sequence of SIX4 compared to IgG (Figure 3(i)). The above experiments demonstrated that FOXA1 can bind to the promoter sequence of SIX4 and affect its expression.

### 3.4. Overexpression of SIX4 Activates the PI3K/AKT Signaling Pathway in CC

**Figure 4:**
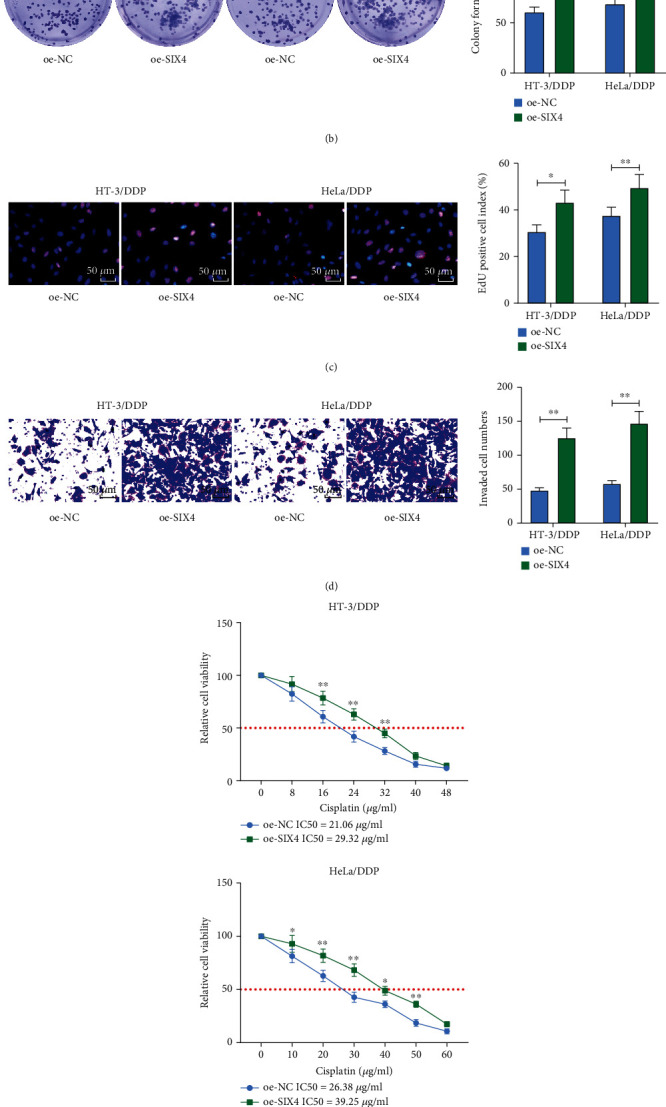
Overexpression of SIX4 activates the PI3K/AKT signaling pathway to promote CC cell growth and chemoresistance to DDP. (a) Detection of SIX4 mRNA expression in HT-3/DDP and HeLa/DDP cells by RT-qPCR. (b) The proliferation of cells measured using colony formation assay. (c) The cell viability measured using EdU assay. (d) The invasive ability of cells determined using Transwell assay. (e) The chemoresistance of cells to DDP measured using MTT assay. (f) The protein expression of p-PI3K, PI3K, p-AKT, and AKT in cells examined using Western blot. Data among multiple groups were analyzed by two-way ANOVA followed by Tukey's post hoc test (^∗^*p* < 0.05; ^∗∗^*p* < 0.01). The cell experiment was independently repeated three times.

To further determine the effect of SIX4 on the growth and drug resistance of CC cells, we overexpressed SIX4 in drug-resistant cells HT-3/DDP and HeLa/DDP and found that its mRNA expression was significantly elevated after overexpression of SIX4 using RT-qPCR (Figure 4(a)). Colony formation assays with EdU staining revealed that the activity of drug-resistant cells was significantly higher after overexpression of SIX4 (Figures 4(b) and 4(c)). The results of the Transwell assay showed a significant higher invasive capacity of drug-resistant cells in the oe-SIX4 group (Figure 4(d)). The chemoresistance of resistant cells to DDP was significantly enhanced after overexpression of SIX4, as evidenced by MTT assay (Figure 4(e)). It has been reported that SIX4 expedited metastasis in colorectal cancer by activating the PI3K/AKT signaling pathway [12]. We performed Western blot assays to measure the PI3K/AKT signaling pathway activation in CC cells overexpressing SIX4 cells. It showed that the extent of PI3K and AKT phosphorylation was significantly increased after promoting the expression of SIX4 (Figure 4(f)). The above results indicate that SIX4 can activate PI3K/AKT signaling, thus promoting CC cell growth and enhancing chemotherapy resistance.

### 3.5. Overexpression of SIX4 Partially Reverses the Repressive Effects of sh-FOXA1 on CC Cells

**Figure 5:**
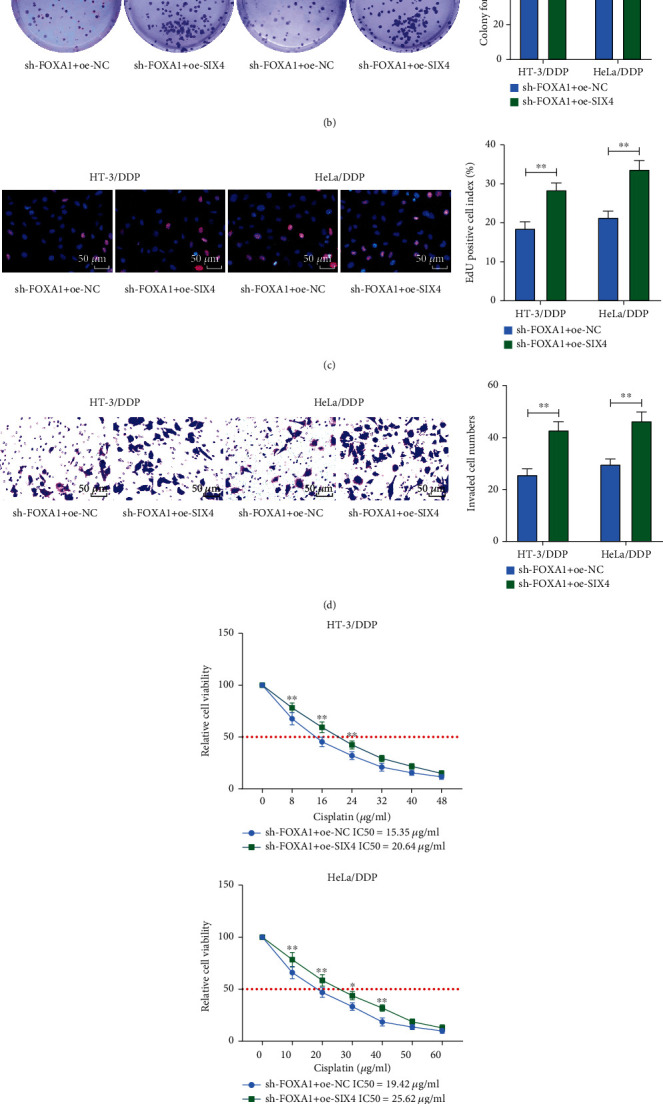
Overexpression of SIX4 partially reverses the inhibitory effect of sh-FOXA1 on CC cell growth and chemoresistance. (a) Detection of mRNA expression of SIX4 in drug-resistant cells after cotransfection by RT-qPCR. (b) The proliferation of cells measured using colony formation assay. (c) The cell viability measured using EdU assay. (d) The invasive ability of cells determined using Transwell assay. (e) The chemoresistance of cells to DDP measured using MTT assay. (f) The protein expression of p-PI3K, PI3K, p-AKT, and AKT in cells examined using Western blot. Data among multiple groups were analyzed by two-way ANOVA followed by Tukey's post hoc test (^∗^*p* < 0.05; ^∗∗^*p* < 0.01). The cell experiment was independently repeated three times.

We overexpressed SIX4 in HT-3/DDP and HeLa/DDP drug-resistant cells with FOXA1 knockdown for rescue experiments. The successful transfection was confirmed using RT-qPCR (Figure 5(a)). We found that further overexpression of SIX4 in drug-resistant cells with FOXA1 knockdown significantly increased the activity of drug-resistant cells by colony formation assay and EdU staining (Figures 5(b) and 5(c)). Transwell invasion assay results showed that overexpression of SIX4 reversed the inhibitory effect of sh-FOXA1 on the ability of drug-resistant cells to invade (Figure 5(d)). We examined the changes in IC50 values of drug-resistant cells to DDP using MTT and observed that overexpression of SIX4 significantly enhanced the chemoresistance of drug-resistant cells to DDP in the presence of sh-FOXA1 (Figure 5(e)). Western blot assay showed that the protein expression of p-PI3K and p-AKT was elevated, and the PI3K/AKT signaling pathway was activated after overexpression of SIX4 (Figure 5(f)). Taken together, overexpression of SIX4 partially reversed the repressing effect of sh-FOXA1 on CC cell resistance to chemoresistance by activating the PI3K/AKT signaling pathway.

## 4. Discussion

DDP has been considered as the primary chemotherapy for locally advanced CC or recurrent cancers, while it should be taken into consideration that the majority of patients treated for persistent and/or recurrent CC represent previous chemo- or chemoradiation failures [13]. Thus, for some patients, the establishment of certain molecular mechanisms impairing the response to DDP might have a significant role in improving poor prognosis. DDP exerts anticancer activity via manifold mechanisms, but its most conventional mechanism remains production of DNA lesions by interacting with purine bases on DNA, followed by activation of several signal transduction pathways [14]. Functional experiments in our study revealed that FOXA1 promoted CC cell resistance to DDP *in vitro* and facilitated tumor growth *in vivo* by upregulating SIX4 expression and activating the PI3K/AKT signaling.

Initially, FOXA1 was characterized to be elevated in CC tissues and cells using data mining, RT-qPCR, and Western blot. Furthermore, silencing of FOXA1 using shRNA inhibited CC cell proliferation, colony formation, and invasion. FOXA1 expression was also significantly higher in colorectal cancer tissues derived from TCGA datasets and was linked to worse prognosis in the R2 database [15]. Hight et al. revealed that reduction of FOXA1 abolished the ability of non-small-cell lung cancer cells to form clonogenicity in vitro (about 4-5 folds of decrease) and tumors in mice [16]. More relevantly, FOXA1 has been reported to inhibit apoptosis and cause abnormal cell proliferation in CC [17]. In addition, increased FOXA1 expression has been recently identified to promote cancer chemotherapy resistance in mice and patients with prostate cancer and breast cancer [18]. FOXA1 upregulation induced genome-wide enhancer reprogramming and activated the HIF-2*α* expression and a prometastatic transcriptional program in endocrine-resistant breast cancer [19]. Upon FOXA1 knockdown in breast cancer cells, Kumar et al. found an increase in doxorubicin and paclitaxel sensitivity and a decline in anchorage independence [20]. Meanwhile, Labbé and Brown identified the association between FOXA1 and castration-resistant prostate cancer [21]. FOXA1 contributed to acquisition of chemoresistance in human lung adenocarcinoma via transactivation of SOX5 [22]. In the present study, we presented evidence showing the sensitizer role of sh-FOXA1 on CC cells to DDP.

FOXA1 has been documented to bind to the promoter of KDM6A and PLOD2, thus promoting their transcription in bladder cancer and non-small-cell lung cancer, respectively [23, 24]. Similarly, in CC, FOXA1 was verified to activate the transcription of LINC00662 and PDK4 [25]. Therefore, we postulated that the regulatory effects of FOXA1 on CC resistance to DDP were elicited through its transcription factor role. As expected, SIX4 was revealed to be a downstream candidate of FOXA1 in CC. SIX4 has been identified as a target of multiple microRNAs in different cancers, including gastric cancer, glioblastoma multiforme, and bladder cancer [26–28]. Intriguingly, Camolotto et al. reported that hepatocyte nuclear factor 4*α* directly repressed SIX4, and SIX4 drove proliferation and differentiation in hepatocyte nuclear factor 4*α*-negative pancreatic ductal adenocarcinoma cells [29]. Here, we revealed that the expression of SIX4 was governed by FOXA1. Moreover, overexpression of SIX4 not only expedited the chemoresistance of CC cells but also reversed the chemosensitivity promoting effects of sh-FOXA1. The tumor-supporting effects of SIX4 were also substantiated in hepatocellular carcinoma, non-small-cell lung cancer, and breast cancer [30–32]. However, its involvement in CC and in chemoresistance is scarcely understood, which highlights the novelty of our study. It has been reported that SIX4 expedited metastasis in colorectal cancer by activating the PI3K/AKT signaling pathway [12]. We similarly found that the extent of PI3K and AKT phosphorylation, which was reduced by silencing of FOXA1, was activated by SIX4 upregulation in CC cells. The interaction between FOXA1 and the PI3K/AKT pathway has also been underscored in hormone sensitivity of breast cancer [33].

## 5. Conclusion

All in all, we identified a novel molecular and functional network in CC cell resistance to DDP that coordinates FOXA1, SIX4, and the PI3K/AKT pathway. The result hinted that FOXA1 knockdown might represent a potential candidate for overcoming CC resistance to DDP. Thus, future work focusing on precise mechanisms that underlie the chemoresistance of DDP could be useful in guiding clinical therapeutic decisions. Besides, the validation of this axis on patient samples might be of great clinical value to develop efficient therapeutic strategy for CC.

## Figures and Tables

**Table 1:** Primer sequences.

Name of primer	Sequences (5′-3′)
FOXA1-F	GCAATACTCGCCTTACGGCTCT
FOXA1-R	GGGTCTGGAATACACACCTTGG
SIX4-F	CGGAGCAAACAGCCAGTTCCTT
SIX4-R	GCTTCCATCTGAAGTGCTTGAGC
GAPDH-F	GTCTCCTCTGACTTCAACAGCG
GAPDH-R	ACCACCCTGTTGCTGTAGCCAA

Note: FOXA1: forkhead-box A1; SIX4: SIX homeobox 4; GAPDH: glyceraldehyde-3-phosphate dehydrogenase; F: forward; R: reverse.

## Data Availability

All the data generated or analyzed during this study are included in this published article.
